# Refractory mogamulizumab-associated rash responding to an oral Janus kinase inhibitor

**DOI:** 10.1016/j.jdcr.2023.06.009

**Published:** 2023-06-17

**Authors:** Carine M. Lama, Miguel A. Hernandez-Rovira, Neha Mehta-Shah, Aaron Russell, Amy C.M. Musiek

**Affiliations:** a Washington University School of Medicine, St. Louis, Missouri; b Division of Oncology, Department of Medicine, Washington University School of Medicine, St. Louis, Missouri; c Division of Dermatology, Department of Medicine, Washington University School of Medicine, St. Louis, Missouri; d Department of Pathology and Immunology, Washington University School of Medicine, St. Louis, Missouri

**Keywords:** cutaneous T-cell lymphoma, Janus kinase inhibitor, mogamulizumab-associated rash, Sézary syndrome

## Introduction

Sézary syndrome (SS) is a rare variant of cutaneous T-cell lymphoma (CTCL), with an age-adjusted incidence of 0.1 per million persons in the United States.^1^ Mogamulizumab (POTELIGEO, Kyowa Kirin, Inc) is an anti-CCR4 antibody that is Food and Drug Administration-approved for the treatment of relapsed or refractory mycosis fungoides and SS. An initial phase 1/2 study evaluating the efficacy of mogamulizumab showed global response rates of 29% and 47% in mycosis fungoides and SS, respectively, with a 95% response rate in the blood.^2^ In a subsequent phase 3 randomized controlled trial, mogamulizumab demonstrated superior progression-free survival compared with vorinostat, another Food and Drug Administration-approved drug for refractory CTCL.^3^ Mogamulizumab-associated rash (MAR) is one of the most common adverse events associated with mogamulizumab therapy, occurring in almost a quarter of patients treated in clinical trials.^3^^,^^4^ Grade 1 rash can typically be managed with high-potency topical steroids, but more severe reactions may require cessation of mogamulizumab and/or treatment with oral corticosteroids.^4^ Herein, we detail a patient with SS treated with mogamulizumab who developed a treatment-refractory rash that ultimately cleared with an oral Janus kinase inhibitor (JAKi).

## Case report

A 62-year-old male presented to the dermatology clinic with a 3-year history of persistent, pruritic rash on the head, neck, trunk, and extremities. Skin biopsies from the left shoulder and left calf revealed an atypical T-cell infiltrate with an elevated CD4:CD8 ratio and partial loss of CD7. Peripheral blood flow cytometry showed a markedly elevated CD4:CD8 ratio of 18.6:1 within T-lymphocytes, with 87% of CD4+ T cells being negative for both CD7 and CD26. T-cell receptor (TCR) gene rearrangement studies were performed on blood and fresh tissue and revealed clonal TCR-γ gene rearrangements of Vg8, Vg9, and JgP2, a common finding in neoplastic T cells. At this point, the patient was diagnosed with SS. He was treated with narrowband UV-B in combination with oral bexarotene for approximately 2 years with only partial clinical response. Given his lack of improvement, the patient was then started on mogamulizumab infusions.

**Fig 1:**
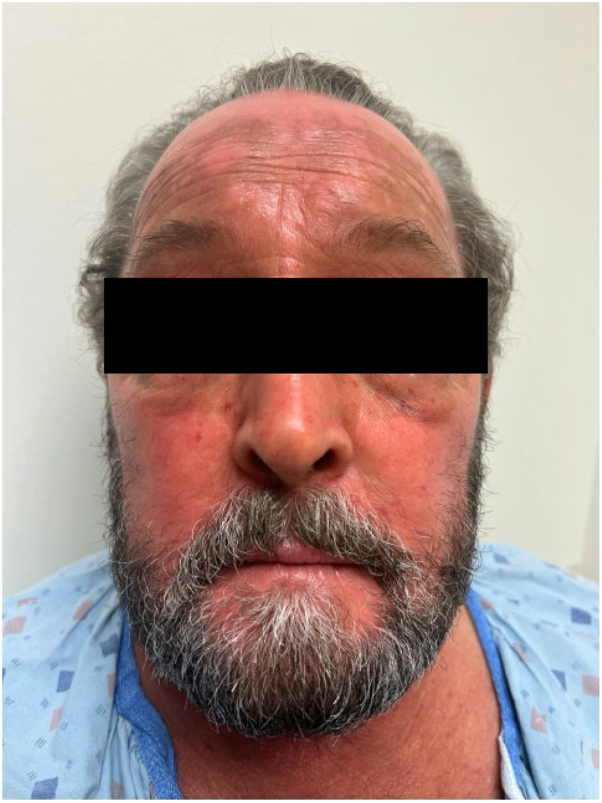
Photodistributed mogamulizumab-associated rash of the face.

**Fig 2:**
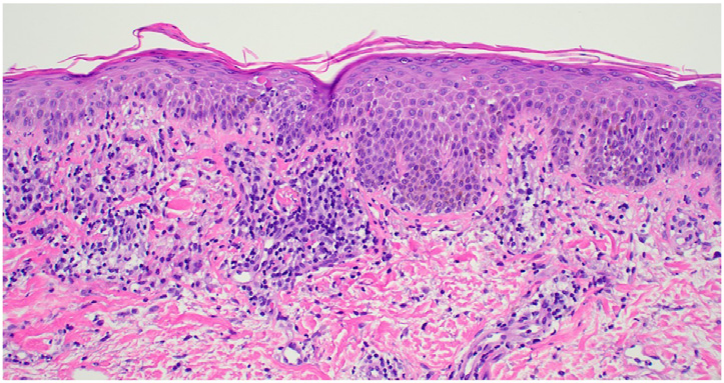
Punch biopsy from back. There is parakeratosis, irregular epidermal hyperplasia, spongiosis, and a superficial perivascular and interstitial inflammatory cell infiltrate that abuts the epidermis where there is patchy basal vacuolar changes and few necrotic keratinocytes. (Hematoxylin-eosin stain; original magnification: ×200.)

**Fig 3:**
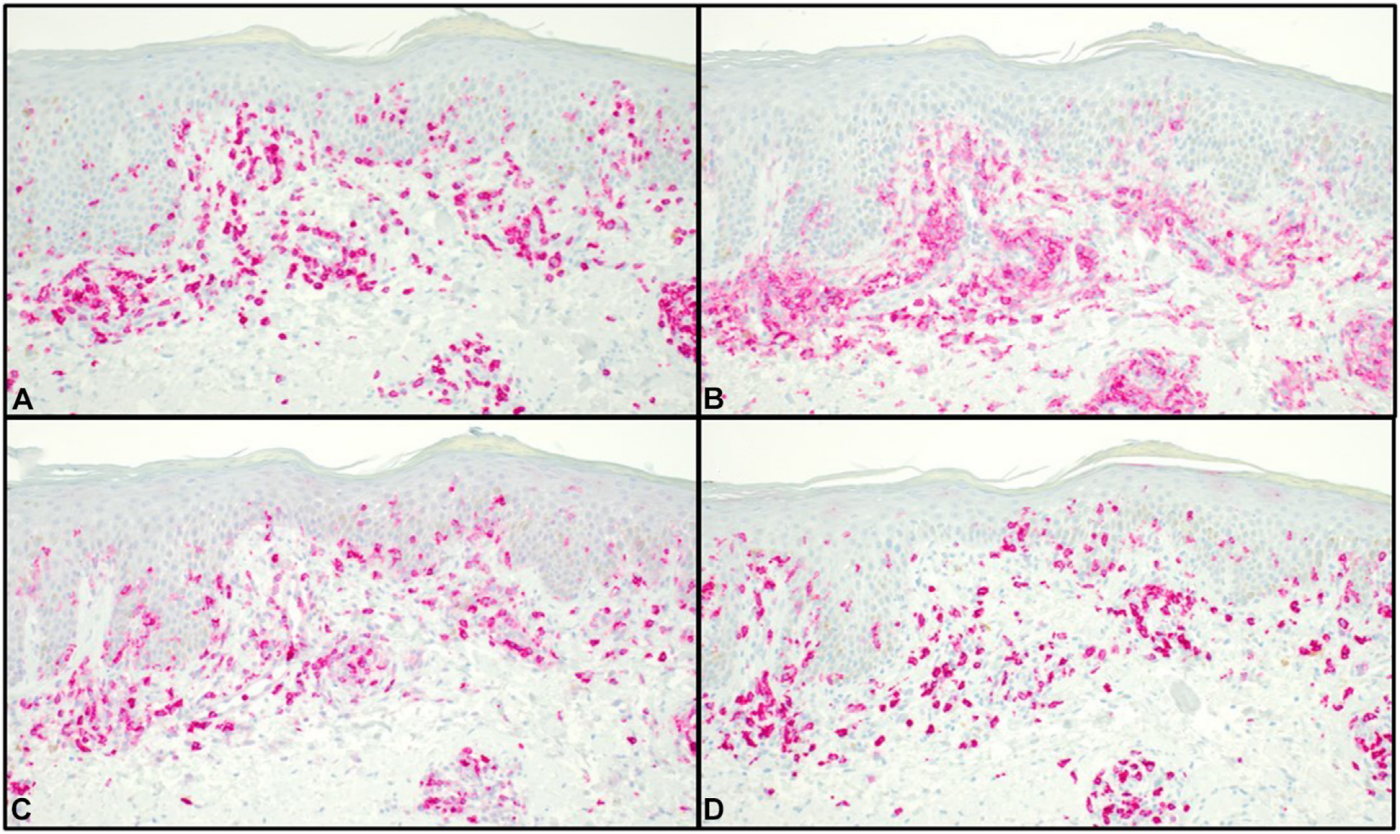
Immunophenotype of intraepidermal T cells in mogamulizumab-associated rash. **A,** There are scattered small CD3+ T-cell within the epidermis. The intraepidermal T cells show a decreased ratio of **(B)** CD4+ to **(D)** CD8+ cells. Normal CD4:CD8 ratio ∼3:1. **C,** There is retained expression of CD7 within the epidermis. (All panels ×200.)

**Fig 4:**
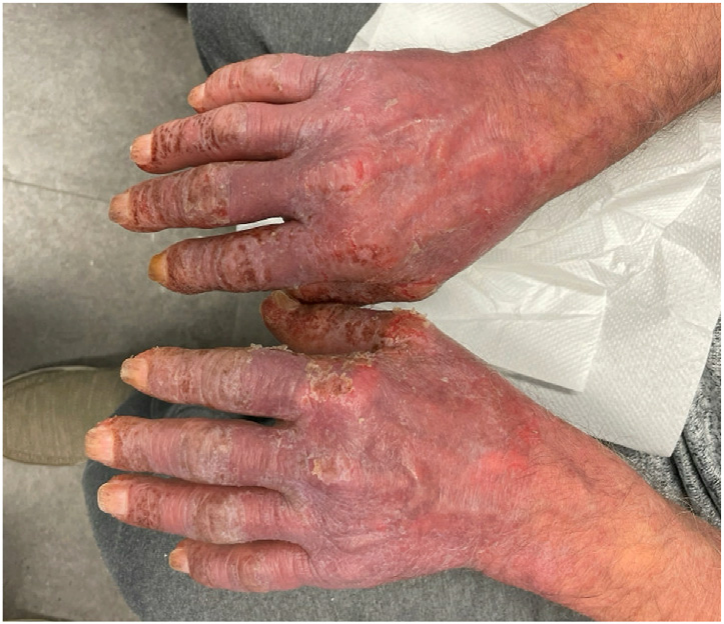
Worsening photosensitive mogamulizumab-associated rash of the hands.

The patient’s rash and pruritus quickly improved on mogamulizumab. In addition, flow cytometry at 42 days showed no evidence of SS in the blood. However, after only 2 cycles of therapy, the patient began to develop a new photodistributed rash on the face, upper portion of the trunk, and extremities (Fig 1). A punch biopsy from the back showed spongiosis and a perivascular, interstitial, and patchy lichenoid inflammatory cell infiltrate consisting of lymphocytes, histiocytes, and rare eosinophils (Fig 2). There were scattered CD3+ lymphocytes within the epidermis that showed a decreased CD4:CD8 ratio (Fig 3). These findings were compatible with MAR. TCR gene rearrangement studies were not performed on the biopsy specimen. Mogamulizumab was discontinued but the rash and pruritus persisted despite treatment with high-potency topical steroids and gabapentin. The rash would acutely worsen with episodes of sun exposure, most severely on the face and dorsal aspect of the hands (Fig 4), even several months after stopping mogamulizumab. Over the next year, the patient was trialed on numerous systemic therapies, including methotrexate, oral corticosteroids, and cyclophosphamide. However, none of these medicines adequately controlled the rash. A repeat punch biopsy from the posterior aspect of the neck showed identical histopathologic and immunohistochemical findings as the prior biopsy, consistent with a MAR. Additionally, flow cytometry continued to show no evidence of SS in the blood despite discontinuation of mogamulizumab.

**Fig 5:**
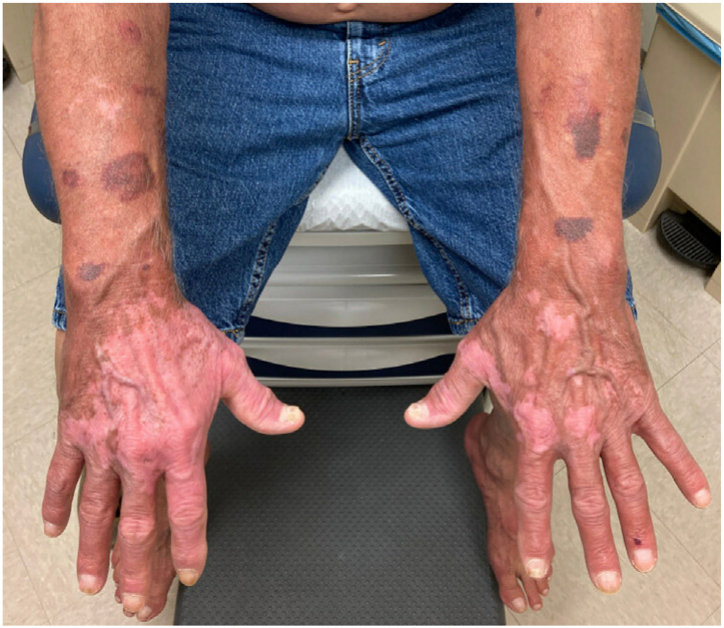
Resolution of mogamulizumab-associated rash secondary to treatment with Janus kinase inhibitor.

After more than a year of failed treatments, the patient was finally started on oral upadacitinib, a selective JAK inhibitor that targets JAK1. Two months after the initiation of upadacitinib, the patient’s rash and pruritus had almost completely resolved with no noted adverse effects. Physical examination at his 2-month follow-up visit showed widespread postinflammatory dyspigmentation but minimal erythema of the skin (Fig 5).

## Discussion

MAR is a common adverse effect of mogamulizumab therapy, occurring in 24% of patients.^4^ Skin biopsy may show various histopathologic patterns, including spongiotic/psoriasiform dermatitis, interface dermatitis, and granulomatous dermatitis.^5^ Multiple reaction patterns may be seen in a single biopsy specimen of MAR, which can help distinguish MAR from CTCL. Immunohistochemical studies can offer additional insight, with MAR showing a normalized or inverted CD4:CD8 ratio within intraepidermal lymphocytes, in contrast to CTCL which typically shows a predominant CD4+ T-cell population within the epidermis.^5^ T-cell clonality studies are another helpful ancillary test. Tissue biopsy specimens from MAR will typically show absence, or exceedingly low levels, of the TCR clones previously identified in lesions of mycosis fungoides/SS.^6^

MAR, although not yet completely understood, has been theorized to be caused by the induction of T-helper 1 (Th1) cell polarization and the reduction of T regulatory cells resulting from the anti-CCR4 action of mogamulizumab.^7^ The appearance of a rash may then be an indicator of drug effectiveness, given previous observations of global clinical response to mogamulizumab treatment coinciding with rash incidence.^8^ Thus, rash development may suggest a shift toward antitumoral Th1 inflammatory action.^8^ Studies demonstrating the decrease in T regulatory cells following JAK inhibition suggest that the therapeutic outcome of treatment of MAR with a JAKi may be due to interruption of Th1 cell function.^9^ In fact, extended treatment with JAKis has been demonstrated to result in polarization away from Th1 toward Th17, which may counteract the Th1 polarization caused by mogamulizumab therapy.^9^ Additionally, the silencing of T-helper cells in both the *in vivo* and *in vitro* settings has been observed, whereby proinflammatory cytokine secretion is inhibited.^6^ This may further act to reduce MAR in patients.

Our initial reasoning for treating refractory MAR with a JAKi came from the documented efficacy of JAKi in CTCL.^10^ Given that MAR may present with a degree of histopathologic similarity to CTCL, and can mimic CTCL clinically,^11^ we believed it to be reasonable to attempt treatment of the refractory MAR with a JAKi. In the current case, upadacitinib was started over a year after the discontinuation of mogamulizumab, and the patient demonstrated a significant reduction in MAR within 8 weeks. Upadacitinib treatment was continued thereafter at the same dose. The use of JAKi as a systemic therapy for MAR is a novel addition to our treatment algorithm that proved to be efficacious in this patient, and as such may inform future treatment of patients with refractory MAR.

## Conflicts of interest

Dr Musiek has received financial compensation through her employment on an advisory board for Kyowa, and as a principal investigator for Kyowa, Pfizer, Aristea, and Bristol Myers Squib. The other authors have no conflicts of interest to declare.
